# Dissecting lung development and fibrosis at single-cell resolution

**DOI:** 10.1186/s13073-019-0645-7

**Published:** 2019-05-24

**Authors:** Donna L. Farber, Peter A. Sims

**Affiliations:** 1 Columbia Center for Translational Immunology, Columbia University Irving Medical Center, New York, NY 10032 USA; 2 Department of Surgery, Columbia University Irving Medical Center, New York, NY 10032 USA; 3 Department of Microbiology and Immunology, Columbia University Irving Medical Center, New York, NY 10032 USA; 4 Department of Systems Biology, Columbia University Irving Medical Center, New York, NY 10032 USA; 5 Department of Biochemistry and Molecular Biophysics, Columbia University Irving Medical Center, New York, NY 10032 USA

## Abstract

Single-cell transcriptome profiling has enabled high-resolution analysis of cellular populations in tissues during development, health, and disease. Recent studies make innovative use of single-cell RNA sequencing (scRNAseq) to investigate mechanisms that allow immune cells to interact with tissue components in the lung during development and fibrotic lung disease.

## Cellular networks in lung development, homeostasis, and disease

The development and maintenance of specialized tissues and organs in the body involves complex cellular and molecular interactions that establish structural integrity, tissue-specific functions, and tissue-intrinsic mechanisms for protection and repair. These coordinated functions are mediated by diverse cell types, including subsets of epithelial and endothelial cells, stromal cells or fibroblasts, and immune cells. In particular, tissue-resident innate and adaptive immune cells, which include tissue macrophages, granulocytes, and lymphocytes, play key roles in sensing and repairing tissue damage and thus mediate in situ protection against environmental and microbial insults. This interaction of immune cells with tissue components is essential for proper development and homeostasis; dysregulation of immune function and/or surveillance can lead to disease manifested by tissue inflammation, fibrosis, or cancer. Defining the nature of these interactions and the mechanisms for their dysregulation is critical to understanding human development, aging, and disease.

The lung is a crucial organ comprised of numerous cell types that mediate respiration and gas exchange. Lung tissues contain multiple immune cell types such as specialized alveolar and interstitial macrophages, dendritic cells, granulocytes, and lymphocytes, including circulating and tissue-resident memory T cells. The lung is constantly exposed to microbial and environmental insults, both pathogenic and non-pathogenic, suggesting that dynamic mechanisms are necessary to maintain tissue integrity. When these processes break down, specific defects in lung repair trigger fibrotic changes to the lung that result in a debilitating syndrome called pulmonary fibrosis, a progressive disease marked by loss of lung function, structural integrity, and respiratory capacity [1]. The course of this disease is irreversible and many patients require lung transplantation, which is the only known cure. Interestingly, it has been shown previously that macrophages play crucial roles in the fibrotic process and exhibit functional alterations in fibrotic lesions, suggesting that fibrosis is associated with defects in both the function of macrophages and their interactions with epithelial cells and fibroblasts [2]. Nevertheless, the mechanisms by which macrophages become altered and promote fibrosis remain unclear.

## Through the single-cell lens

Recent technological advances have enabled the gene-expression profiles of individual cells to be measured by single-cell RNA sequencing (scRNAseq), providing a new opportunity to define the cell types and molecular pathways that are involved in tissue homeostasis and disease with high precision. scRNAseq has elucidated cellular compositions, heterogeneity, and developmental and activation states in diverse systems [3, 4]. Unlike conventional bulk methods, scRNAseq enables the identification of rare cell types and is particularly amenable to studies of populations undergoing asynchronous transitions. In human bronchial and mouse tracheal epithelium, scRNAseq has been used to identify new cell subsets and populations that are potentially involved in airway disease [5, 6]. The rapid increase in scalability of scRNAseq has given rise to large datasets, further necessitating the development of innovative approaches for data analysis that improve the identification of cell subsets, differentiation and functional states, and moving beyond cell clustering into dissecting cell–cell interactions and functional pathways. Thus, obtaining new biological and mechanistic insights from scRNAseq data requires new computational approaches that are tailored to the specific scientific question at hand.

Three recent studies focusing on the lung applied cutting-edge scRNAseq, along with the development or application of novel computational analyses, to investigate the cell-state transitions involved in the development of lung fibrosis in both mouse models and humans [7, 8] and to delineate the cell–cell interactions involved in establishing lung-resident macrophages during normal lung development [9].

## Cell state transitions in lung fibrosis

The study by Aran et al. [7] used scRNAseq to profile mouse lung cells in steady state and in well-characterized models of pulmonary fibrosis, including the bleomycin-induced model of lung injury, which triggers widespread epithelial damage and lung fibrosis, and an alternative model involving telomere dysregulation. Unbiased cell-type identification for scRNA-seq is challenging, particularly in diseased tissues where pathogenic responses may distort a canonical phenotype. The authors therefore developed an algorithm called SingleR that performed cell-type annotation by systematic comparison of scRNA-seq profiles to reference data [7]. They identified a novel subpopulation of monocytes that exhibited markers, including *Cx3cr1*, *Ccr2*, and MHC class II genes, that were associated with transition to the alveolar macrophage phenotype that occurs in lung fibrosis in these animal models. They further demonstrated that this subset of macrophages was the prime source of platelet-derived growth factor–AA (Pdgf-aa), which is involved in promoting fibroblast proliferation, and that ablation of this subpopulation in mice with conditional deletion of CX3CR1-expressing cells mitigated fibrosis. These findings, elucidated by unbiased scRNAseq analysis of cellular populations during the peak fibrotic response, revealed the precise identity of the pathogenic infiltrating population during fibrosis.

Animal models enable elegant functional validation and elucidation of developmental time courses in an isogenic background, but analysis of patient specimens with the resolution of scRNA-seq will be crucial to identifying the macrophage subpopulations that are involved in disease pathogenesis and to deriving potential therapeutic targets. Reyfman and colleagues used scalable methods for scRNA-seq for unbiased analysis of lung biopsies from patients with fibrosis and of healthy lung tissue from transplant donors [8]. Like Aran et al. [7], Reyfman et al. [8] analyzed scRNAseq data in conjunction with reference datasets for known immune cells, epithelial cells, and fibroblasts. This identified fibrosis-specific subpopulations of macrophages that exhibit a pro-fibrotic phenotype along with specific fibrosis-induced changes in alveolar epithelial cells [8]. Although scRNAseq is invaluable for marker discovery, many transcripts go undetected (an issue known as transcript ‘drop out’), which can compromise measurements of cellular composition that are based on a small set of genes. These studies demonstrated how in situ RNA hybridization in patient biopsies could be used to improve estimates of cellular composition that are based on markers derived from scRNAseq [7, 8].

## Cell–cell interactions in lung development

In addition to identifying cellular states for lung fibrosis, data obtained from scRNAseq can also provide insights into complex cell–cell interactions. The study by Cohen et al. [9] reports an innovative approach to analyzing scRNAseq profiles using protein–ligand interaction networks to infer cell–cell interactions in the developing lung. They analyzed the murine lung with scRNAseq using unbiased sampling of the cellular population at seven stages of embryonic and post-natal development. In addition to inferring cell subsets and states using graph-based clustering, they leveraged published ligand–receptor pairs to build an interactome between ‘meta-cells’ that would elucidate crosstalk between groups of cells with similar expression profiles. The resulting interaction network suggested that lung-resident basophils, a relatively rare cell type, were highly connected to both immune and non-immune cells in the lung. Validation studies by co-culture and selective ablation of basophils revealed a previously undefined, essential role for cells of this type in the development of the steady-state alveolar macrophage phenotype. This role involves basophil-specific IL-33 production which promotes an anti-inflammatory gene-expression program, including enhanced expression of *Ccl17*, *Arg1*, and *Itgax*, in macrophages. These findings establish the power of scRNAseq data in revealing critical interacting pathways involving multiple cell types in complex tissue environments. As repair processes can recapitulate developmental processes in tissues, it will be interesting to analyze the scRNAseq data in lung fibrosis, a disease associated with altered or defective repair, to determine whether basophils are involved in the disease process.

## Conclusions

Unbiased application of scRNAseq to sample the cellular population within a tissue produces high-dimensional data from which both molecular and cellular interactions can be inferred. The studies discussed here apply scRNAseq to analyze dynamic processes in the lung that are involved in development and repair. These approaches have revealed new insights into the identity of innate immune cells, including macrophage subsets and basophils, that mediate lung-cell development and are implicated in dysregulated repair processes in fibrosis. As we accumulate data from different tissues in distinct developmental and pathological contexts, we will be able to define the molecular alterations in specific cell types and locations within a tissue that are associated with and predictive of disease. We anticipate that this approach will be essential to the development of precision therapeutics with high molecular and cellular specificity.

## Abbreviation

scRNAseq: Single-cell RNA sequencing
